# Puzzle-Solving Activity as an Indicator of Epistemic Confusion

**DOI:** 10.3389/fpsyg.2019.00163

**Published:** 2019-01-31

**Authors:** Amaël Arguel, Lori Lockyer, Kevin Chai, Mariya Pachman, Ottmar V. Lipp

**Affiliations:** ^1^Department of Educational Studies, Macquarie University, Sydney, NSW, Australia; ^2^Faculty of Arts and Social Sciences, University of Technology Sydney, Sydney, NSW, Australia; ^3^Curtin Institute for Computation, Curtin University, Perth, WA, Australia; ^4^Department of Educational Psychology and Learning Systems, Florida State University, Tallahassee, FL, United States; ^5^School of Psychology, Curtin University, Perth, WA, Australia

**Keywords:** confusion, learning, epistemic emotions, problem solving, interaction analytics

## Abstract

When students perform complex cognitive activities, such as solving a problem, epistemic emotions can occur and influence the completion of the task. Confusion is one of these emotions and it can produce either negative or positive outcomes, according to the situation. For this reason, considering confusion can be an important factor for educators to evaluate students’ progression in cognitive activities. However, in digital learning environments, observing students’ confusion, as well as other epistemic emotions, can be problematic because of the remoteness of students. The study reported in this article explored new methodologies to assess emotions in a problem-solving task. The experimental task consisted of the resolution of logic puzzles presented on a computer, before, and after watching an instructional video depicting a method to solve the puzzle. In parallel to collecting self-reported confusion ratings, human-computer interaction was captured to serve as non-intrusive measures of emotions. The results revealed that the level of self-reported confusion was negatively correlated with the performance on solving the puzzles. In addition, while comparing the pre- and post-video sequences, the experience of confusion tended to differ. Before watching the instructional video, the number of clicks on the puzzle was positively correlated with the level of confusion whereas the correlation was negatively after the video. Moreover, the main emotions reported before the video (e.g., confusion, frustration, curiosity) tended to differ from the emotions reported after the videos (e.g., engagement, delight, boredom). These results provide insights into the ambivalent impact of confusion in problem-solving task, illustrating the dual effect (i.e., positive or negative) of this emotion on activity and performance, as reported in the literature. Applications of this methodology to real-world settings are discussed.

## Introduction

Learning a technique, understanding a situation, solving a problem, and more generally, developing knowledge about a topic, are examples of complex cognitive activities. These learning tasks involve specific cognitive processes in order to select, to organize, and to integrate different pieces of information into a mental model (Mayer, 2009). In addition to the cognitive engagement of such tasks, learners are also likely to experience emotions when attempting to understand complex content portrayed in instructional environments (Um et al., 2012). The emotions that occur during learning are sometimes called *epistemic emotions* because they are directly caused by the cognitive processing of information presented as part of a learning task (Pekrun and Linnenbrink-Garcia, 2014). D’Mello et al. (2014) distinguished nine epistemic emotions likely to occur when learning from a digital environment, for which they gave the following definitions (p. 158). Anxiety as being nervous, uneasy, apprehensive, or worried; boredom as being weary or restless through lack of interest; confusion/uncertainty as a noticeable lack of understanding and being unsure how to proceed; curiosity as a desire to acquire more knowledge or to learn the material more deeply; delight as a high degree of satisfaction; engagement/flow as a state of interest that results from involvement in an activity; frustration as dissatisfaction or annoyance from being stuck; surprise as a state of wonder or amazement; and neutral as having no apparent emotion or feeling. Typical emotions experienced during a learning task that have significant impact on learning outcomes include, confusion, frustration, boredom, or engagement (D’Mello and Graesser, 2012). For teachers, educators, and designers of learning environments, considering the epistemic emotions that occur during learning can offer an interesting opportunity to improve learning experiences and outcomes.

Among the epistemic emotions generally encountered in learning, confusion is unique since it can produce either negative or positive learning outcomes, depending on the situation. Confusion is generally understood to be the emotional expression of a *cognitive disequilibrium*, which is produced when inconsistent pieces of information need to be integrated together (Graesser and D’Mello, 2012). This inconsistency is generally provoked when different elements of the information from the environment cannot be easily combined in a meaningful way, or for example, when the response from a system is not compliant with the predictions that a learner has made (D’Mello and Graesser, 2006). In game-based environments, for instance, cognitive disequilibrium can occur when players perform actions that produce unexpected results (Lehman et al., 2012). Cognitive disequilibrium can also appear when instructional content bring new information that is incompatible with the prior knowledge of learners on the topic. In this case, learners need to update their initial conceptions of the topic in order to reduce their confusion, which is generally reported as a mildly negative emotion (Kort et al., 2001; Limón, 2001). When doing this, they are also likely to become more engaged in the task, while producing an effort to reduce confusion (D’Mello et al., 2014). Conversely, if confusion is too strong and learners cannot quickly resolve it, they tend to become disengaged and they can subsequently experience other emotions, such as boredom. In this case, learners would most likely experience an overall negative feeling about the activity, and their disengagement can even lead to the ultimate outcome consisting of giving up (Baker et al., 2010). Further, studies have showed that the effects of boredom could be particularly persistent and harmful to learning as it can prevent students to subsequently engage in other learning tasks (Baker et al., 2010; D’Mello and Graesser, 2011). Consequently, understanding the dynamic of the succession of epistemic emotions that occur during learning, as well as the capability to detect some of them, can be crucial for improving learning environments and learners’ experience.

In learning situations, the identification of a learner’s emotional responses, including those of confusion, can be challenging. The detection of confusion is particularly difficult because the experience of it can dramatically vary among individuals for the same learning situation. These individual differences can be caused for example by the variability of levels of prior knowledge or their motivation to learn (Sullins and Graesser, 2014). In face-to-face learning situations, such as in the classroom, teachers can fairly notice some emotional states, including confusion, from the facial expressions of their students (Lipson, 1992). However, when learning takes place in digital environments, the detection of confusion represents a significant challenge because learners may not be co-located with teachers while engaging with learning tasks via computer or other devices (Arguel et al., 2017). Thus, learners may not have ready access to teachers who might monitor their learning progress or peers whom they might consult with to check their understanding. Within these digital contexts, which are increasingly used in formal and informal learning settings, it is crucial to elaborate methodologies that help to detect epistemic emotions, and particularly confusion (Calvo and D’Mello, 2010). The capability of a real-time detection of emotions could allow learning environments to respond to students’ emotional states with the provision of help, feedback, or even the suggestion of adopting relevant self-regulated learning strategies (Arguel et al., 2018; Lodge et al., 2018). However, current equipment and methodologies used to detect confusion and other emotions in laboratory studies, such as eye tracking, physiological and/or behavioral activity, are often complex and not easy to deploy in real-world applications (Arguel et al., 2017). What is needed to have practical impact for the effective design of learning environments is to rely on data that could be collected with equipment that is already currently used for education.

In digital environments, most of the data readily available are generated by human-computer interaction. Recently, methodologies inspired by research on learning analytics (Siemens, 2013) have been considered for a real-time detection of emotions in digital environments (Rienties and Rivers, 2014; Arguel et al., 2019). With this approach, patterns of participants’ behavior are captured from human-computer interaction, that is all the user actions performed with interface elements such as mouse, keyboard, touchscreen, etc. However, each situation requires identifying data and patterns of actions that are the most appropriate in order to identify the occurrence of certain emotions.

This article reports a study in which it was assumed that participants’ actions while solving a game consisting of visual logic puzzles could reflect the strategies employed, and also serve as indicators of the level of cognitive disequilibrium and confusion (Graesser et al., 2005). More precisely, it was foreseen that high levels of confusion would have been linked to a behavior consisting of searching widely for solutions, hence producing an increase of the width of the distribution of participants’ action locations on the puzzles.

## Experimental Study

The study reported in this paper aimed to test a methodology allowing remote and real-time detection of epistemic emotions, notably confusion. For the sake of collecting data usable in real-world applications, the study was specifically designed to avoid relying on laboratory methodology and equipment to detect indicators of participants’ behavior and emotions. The experimental material of the study was a logic puzzle game that was assumed to create some confusion with participants. The objective was to assess at what extent the solving strategies that the participants employed could reveal a relationship with their self-reported emotional experiences.

Logic puzzles exemplify the complexity of learning mathematics, which involves developing an understanding of sets of formal rules, various techniques, and apply strict procedures to be able to resolve problems. For example, one way to learn how to solve a system of linear equations consists in expressing the equation in the form of a visual augmented matrix and to perform a row reduction, also known as the mathematical method of Gaussian elimination (Anton, 2013). While learning this technique can be difficult for students, due to its formalism and its abstractness, the use of visual logic puzzles can represent a helpful introduction. For the study reported in this paper, a visual logic puzzle in the form of a digital game called “Lights Out” was used as learning task to help students understand the Gaussian elimination technique (Mulholland, 2011). However, because solving complex puzzles such as Lights Out is a cognitively demanding task, it can intrinsically generate confusion (D’Mello et al., 2014). In this case, confusion would not be related to a pure learning activity, but to a problem-solving task. Nevertheless, we assumed that the cognitive processes at the origin of confusion are similar: a lack of appropriate prior knowledge while trying to solve the puzzles would cause unexpected results, which is likely to create cognitive disequilibrium (Graesser et al., 2005). Hence, it was hypothesized that improving prior knowledge, with the provision of a solving method presented with an instructional video, should perceptibly reduce the level of confusion.

The research question we addressed was to test if participants’ interaction with the puzzles could be indicators of the emotional state of confusion. Adapted to the experimental material involved in our study, the hypothesis was based on a broader exploration strategy for participants experiencing cognitive disequilibrium (Graesser et al., 2005). Indeed, at some stages of the puzzle-solving task it was expected that novice puzzle-solvers engaged in an exploratory activity, based on trial-and-error, with a variety of moves in order to find an adequate solving strategy (Gick, 1986). We assumed that this solving behavior could be an indicator of relatively high levels of confusion. Conversely, participants with higher expertise should experience less cognitive disequilibrium and, consequently, should report lower levels of confusion (Sullins and Graesser, 2014). In this case, the interaction with the puzzles could likely be more systematic, centered on proximal cells of the puzzles and following a predefined strategy.

In addition to the consideration of confusion levels during puzzle-solving activity, our study also intended to observe the occurrence of other epistemic emotions. According to the literature, the transitions between the epistemic emotions occurring during complex cognitive activities seem to follow a certain mechanism (D’Mello and Graesser, 2012; D’Mello et al., 2014). From the observation of the transitions of emotions measured at regular interval, a theoretical framework called the *model of affect dynamics* has been defined (D’Mello and Graesser, 2012). In the study at the origin of this model, participants generally reported four primary emotions (i.e., engagement, confusion, frustration, and boredom) and the transitions between these emotions were observed mainly between proximal emotions as displayed in the model:

Engagement⇔Confusion⇔Frustration⇔Boredom

Transitions were hence frequently observed from confusion to frustration and then to boredom, or from engagement to confusion, but not between confusion and boredom, engagement and frustration or engagement and boredom. However, it is important to note that in this study, the induction of confusion was controlled and provoked at frequent intervals by responses from an intelligent tutoring system in the learning environment. The authors did not test the effect of an absence of feedback from the system, which could likely produce different emotional transitions. In our study, only the novelty of the puzzles was manipulated to induce confusion. Hence, we also hypothesized that the emotion called engagement could fade away after a period of time without experiencing confusion, and leading consequently to boredom. If this result was observed, it could refine the model of affect dynamics and highlight another explanation of the positive role of confusion, when it helps sustain engagement of students into a cognitively demanding task.

## Materials and Methods

### Participants

To achieve a statistical power of 80% and a level of significance of 5% (two-tailed) for within-subject study design and a negligible dropout rate, we estimated that our study required a minimal sample size of 25 participants. We based this estimation on results from a previous study that used a similar tool for rating confusion at different stages of a problem-solving task (Pachman et al., 2016). In this study, the confusion scores observed in the within-subject comparison were, respectively *M_1_* = 8.69; *SD_1_* = 1.93 and *M_2_* = 6.85; *SD_2_* = 3.05.

Thirty-one volunteer participants were recruited on the campus of a large metropolitan university in Sydney, Australia. The study was advertised on campus and informed consent was obtained from volunteer participants prior to the testing session. Participants were compensated $15 (AUD) for 1 hour of their time participating in the study (none exceeded 1 h of testing). The average age was of 21.8 years, ranging from 18 to 30 years (*SD* = 2.79), and 25.8% of the sample was female participants. The eligibility criteria were to be aged above 18 years and to have normal vision without or after correction. All the participants were university students enrolled in various courses from Social Sciences (e.g., Psychology, Linguistics, Education, History, Geography), Commerce (e.g., Accounting, Applied Finance, Economics), or Arts and Humanities (e.g., Creative Writing, Indigenous Studies, French). Consequently, it was assumed that the participants were likely to have only limited mathematical knowledge. The mathematical method of Gaussian elimination is included in the senior secondary Australian curriculum only for students choosing the optional subject *Specialist Mathematics* (Australian Curriculum, Assessment and Reporting Authority, 2016). It is unlikely student enrolled in Human Sciences, Social Sciences, Business Administration and Art will have completed this specialist course. None of the participants were enrolled in courses such as Mathematics, Physics, or Engineering. Moreover, none had reported any experience of playing any version of Lights Out game prior to the study.

### Material

The material used in the study was based on Lights Out Deluxe, an electronic game by Tiger Toys^TM^, released in 1996. A computer emulation of the game was developed as a JavaScript Web application and presented to participants on a 19-inch touchscreen display (LG^TM^ T1910) allowing naturalistic interaction needed to solve the puzzles. In order to prevent the arm of participants to interfere with the eye tracker beam located at the bottom center of the screen, the puzzles were displayed on a side of the screen corresponding to participant’s dominant hand. Consequently two versions of the material were developed in order to address the potential variability of handedness of participants. However, it appeared finally that all the participants of the study were right-handed, so only the right-handed version of the puzzles was actually used.

**FIGURE 1:**
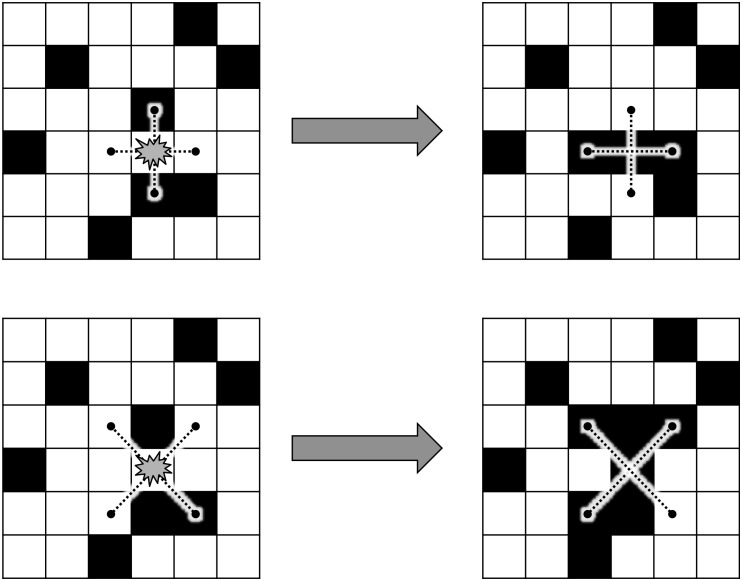
Example of an action on the Type-+ (top) and the Type-x (bottom) puzzle.

The puzzles were comprised of a 6-by-6 matrix of cells colored either yellow or black. When the game started, a random-like pattern of these colored cells was displayed. The goal of the game was to switch all the cells to black, preferably with the least number of moves. Pressing on a cell switched its color but also the color of some of the adjacent cells. There were two versions of puzzles regarding which adjacent cells were affected by a press: in the “Type-+” puzzles, only the cells located to the right/left and top/bottom of the pressed cell are affected, whereas in the “Type-x” puzzles, only the diagonally adjacent cells (i.e., the cells located at the corners of the pressed cell) were impacted (see Figure 1).

### Design

The study was based on a within-subject design, with multiple counter-balanced trials. Participants were initially randomly allocated to one of the puzzle type (i.e., “x” or “+”). Then, participants received written instructions asking to solve as many puzzles as possible within 7-min sequences. When a puzzle was correctly solved (i.e., when all the cells were turned black), the system displayed a congratulation prompt to the participant, inviting them to solve the next puzzle. Then, a new puzzle consisting of a new layout of black and yellow cells was displayed. After the initial 7-min solving sequence (i.e., pre-video sequence), participants watched a short instructional video presenting an effective strategy to solve the puzzle. Each video (i.e., according to puzzle types) showed a step-by-step procedure for solving the puzzles with a video screen capture accompanied by oral commentaries. The solving strategy presented, known as “light chasing,” consisted of solving the top row of puzzle first, then solving row after row until reaching the bottom row. Then, when lights were displayed on the bottom row only, participant had to turn on light(s) from the first row according to a diagram indicating which top-row lights to reactivate. Finally, a second iteration of light chasing from top to bottom turned all the lights off and solved the puzzle. This technique was similar to the Gaussian elimination method (Mulholland, 2011). The length of the video was of 3 min 30 s for the Type-x puzzle and of 2 min 35 s for Type-+.

To ensure that participants were exposed to all the parts of the video and processed them correctly, they were offered the opportunity to watch the video a second time, on demand. After watching the video, participants performed another 7-min solving sequence with the same puzzle type, which is referred to as the post-video sequence. During the post-video solving sequences, a picture of the diagram was presented next to the puzzle as a reference to the solving technique.

**FIGURE 2:**
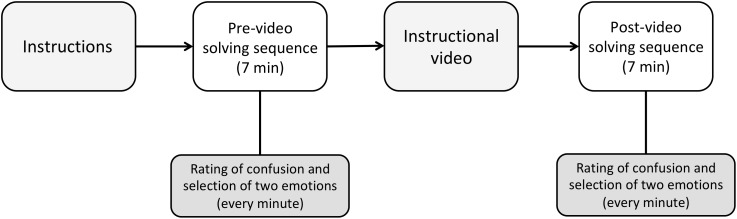
Study protocol (repeated twice).

In the second half of testing, participants performed the same pair of sequences (i.e., pre- and post-video), but with the second type of puzzle (either “x” or “+,”depending on the puzzle that was initially allocated). Altogether, each participant at the end of testing had solved the puzzles in four sequences, with the combination of the variables pre-/post-video and type of puzzle (see Figure 2).

A Tobii X2-60 eye tracker and a computer running the acquisition software Tobii Studio 3.2 was used for the purpose of recording participants’ interaction with the puzzles (i.e., the user’s actions on the touchscreen). By default, this instrumentation also collected gaze trajectories. However, although these data were recorded and stored, they have not been analyzed because the aim of the study was to focus on variables that are readily available in real-world applications. The benefit of not relying on eye-tracking data captured in a laboratory for developing methods to detect and/or measure confusion could be more readily applied in digital environments such as learning management systems and other websites.

During each sequence, the solving activity was interrupted every minute and participants were asked to answer an online survey presented on a tablet computer that the experimenter provided. In this survey, participants were asked to rate their level of perceived confusion by dragging the cursor of a 0–100% visual analog rating scale (Reips and Funke, 2008). In addition, they selected the two most prominent emotions they experienced for each 1-min interval among a list of nine emotions presented in alphabetical order along with their definitions (i.e., anxiety, boredom, confusion, curiosity, delight, engagement, frustration, neutral, and surprise), as per D’Mello et al. (2014). This was done with the tablet computer by a “drag and drop” gesture consisting of moving names of emotion from the list to two boxes labeled first and second emotion. Overall, with four solving sequences composed of seven intervals, each participant had in total 28 occasions to produce data including the rating of confusion and the selection of epistemic emotions. Once all the solving sequences were completed, participants completed a survey on their demographics.

## Results

The dependent variables were the solving performance, the rating of confusion and the selection of other epistemic emotions, and also the on-screen activity of participants when solving the puzzles. The solving performance was assessed with the number of puzzles solved within each of the four 7-min solving sequences. The rating of confusion and the selection of two emotions among a list were performed every minute during the solving sequences. Finally, the solving activity was captured from continuous recording of the time and spatial coordinates of each action produced by the participants on the touchscreen, that we have called clicks. Statistical test significance was assessed at the *p*-value level α = 0.05, except when two hypotheses were tested with the same dataset (i.e., the solving performance and the rating of confusion) for which a Bonferroni correction was applied, resulting in an adjusted *p*-value of α/2 = 0.025.

### Solving Performance

The number of puzzles solved within each of the 7-min solving sequences was the indicator of performance used in the study (see Table 1). It seemed that watching the instructional videos appeared to be an effective way to improve puzzle-solving performance; the number of puzzles improved after watching each of the instructional videos.

**Table 1:** Solving performance, expressed in average number of puzzles solved within each 7-min sequence.

Puzzle version	Type-+			Type-x		
		
	*M*	*SD*	*n*	*M*	*SD*	*n*
Pre-video	0.74	1.21	31	0	0	31
Post-video	10.7	5.24	31	7.68	4.64	31


In order to deal with missing values, we performed a linear mixed model statistical analysis (LMM) with Type of puzzle and Pre-/post-video as fixed effects and Participants as a random effect. The results showed a significant effect of the type of puzzle on solving performance, β = 1.88, *SE* = 0.64, *t*(121) = 2.92, *p* < 0.004, the participants solving the Type-+ puzzles significantly better by than Type-X puzzles. The statistical analysis also confirmed that watching the videos had an effect on solving performance, β = 8.82, *SE* = 0.64, *t*(121) = 13.6, *p* < 0.0001, which indicated that the number of puzzles solved after watching the videos was higher than the number of puzzles solved in the sequences preceding the videos.

### Rating of Confusion

During all the four solving sequences, participants were asked every minute to self-report the level of confusion they were experiencing on 0–100% visual analog rating scales (see Table 2). To test the effect of watching the videos and of the type of puzzle, we performed a 2 × 2 repeated-measures analysis of variance (rANOVA) on the ratings of confusion.

**Table 2:** Average rating of confusion across the sequences.

Puzzle version	Type-+			Type-x		
		
	*M*	*SD*	*n*	*M*	*SD*	*n*
Pre-video	48.4	26.4	31	53.4	27.7	31
Post-video	14.4	22.1	31	21.2	25.9	31


We found a significant effect of watching the videos, *F*(1,30) = 81, *p* < 0.0001, η^2^ = 0.73, indicating that overall, the average rating of confusion was lower in the sequences following the instructional video (*M_post_* = 17.85, *SD_post_* = 24.16) than in the sequences completed before the video (*M_pre_* = 50.94, *SD_pre_* = 26.99). This result suggested that the knowledge of the solving methods depicted in the videos had an impact on the reduction of the level of confusion.

At the adjusted *p*-value level of 0.025, the statistical analyses did not revealed an effect of the type of puzzle on confusion ratings, *F*(1,30) = 4.61, *p* = 0.04, η^2^ = 0.13. In addition, neither interaction between the factors “watching the videos” and “type of puzzle” (*F* < 1) nor any simple effect of the intervals within the solving sequences was statistically significant. The latter observation can suggest a relatively steady level of rating of confusion within each solving sequence.

### Relationships With Performance

We carried out analyses to explore the relationships of solving performance with participants’ activity and levels of self-reported confusion. Because the performance was relatively low in the pre-video sequences, and even null with the type-x puzzle, they were not considered and only the post-video sequences have been analyzed.

**FIGURE 3:**
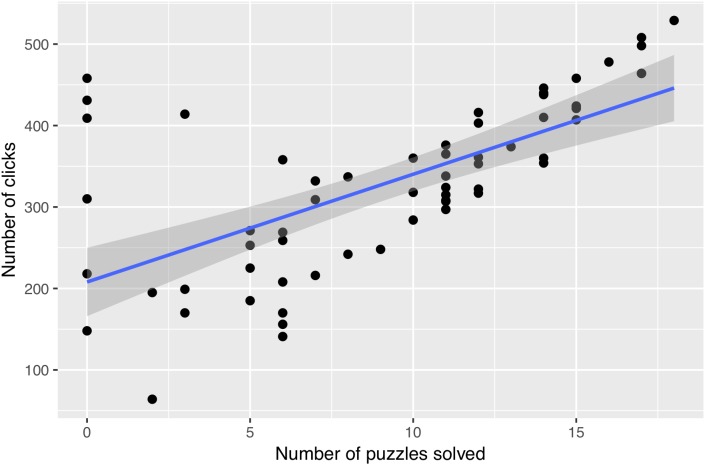
Relationship between activity in number of clicks and performance (post-video).

In order to assess the relationship between the levels of activity of participants (i.e., the number of clicks on puzzles) and performance (i.e., the number of puzzles solved), we performed a regression analysis. Since two solving sequences per participant were involved in the analysis with the two types of puzzle, we used a procedure for repeated-measures correlation as per Bakdash and Marusich (2017). We observed a strong positive correlation between the activity of participants and their performance, *r_rm_*(30) = 0.874, *p* < 0.0001 (see Figure 3). Therefore, it seemed that in the post-video sequences, participants who solved many puzzles also showed a higher level of activity with the production of numerous clicks while solving the puzzles.

**FIGURE 4:**
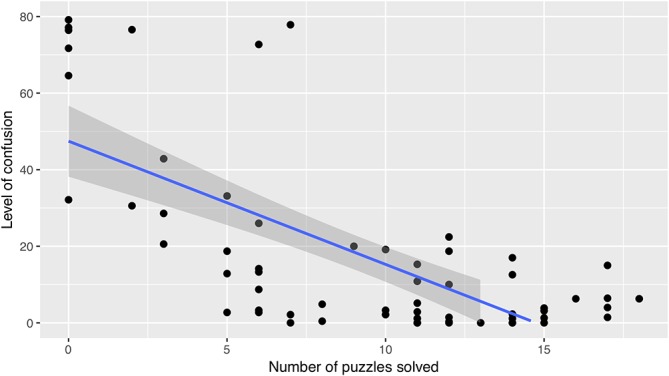
Relationship between average level of confusion and performance (post-video).

A similar analysis was performed to explore the relationship between the levels of self-reported confusion and performance. We observed a strong negative correlation between the two variables (see Figure 4), *r_rm_*(30) = -0.858, *p* < 0.0001. This correlation suggested that the level of confusion tended to be lower when solving performance improved.

### Selection of Other Epistemic Emotions

**FIGURE 5:**
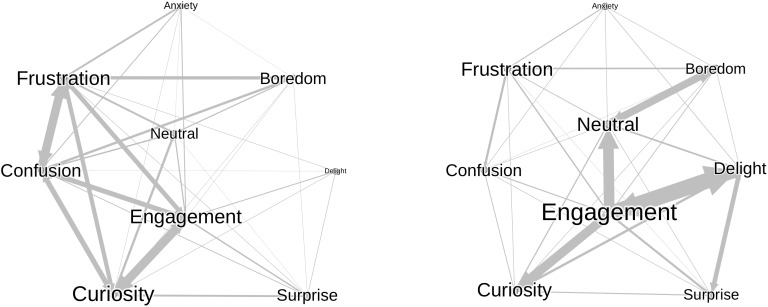
Network graph of selected emotions before (left) and after the instructional video (right).

Along with rating their level of confusion every 1-min during the solving sequences, participants were also asked to choose, within a list of nine, the two most relevant emotions for reflecting their experience. A visualization of these data was generated with relationship network graphs (see Figure 5). Interestingly, participants were capable of selecting two emotions of different valence, like for example Frustration and Engagement, to report their emotional experience at each interval. Moreover, when comparing the networks of affective states before and after the instructional video, it appeared that the most prevalent emotions differed notably across the solving sequences. Indeed, the first emotions reported during the pre-video solving sequences were Engagement, Curiosity, Frustration, and Confusion, whereas the first emotions were Engagement, Curiosity, Delight, and Neutral during the post-video sequences. This result seemed to confirm the efficiency of the experimental material for initially inducing confusion (at pre-video sequences), and the efficacy of the instructional videos for reducing the level of confusion (at post-video).

**FIGURE 6:**
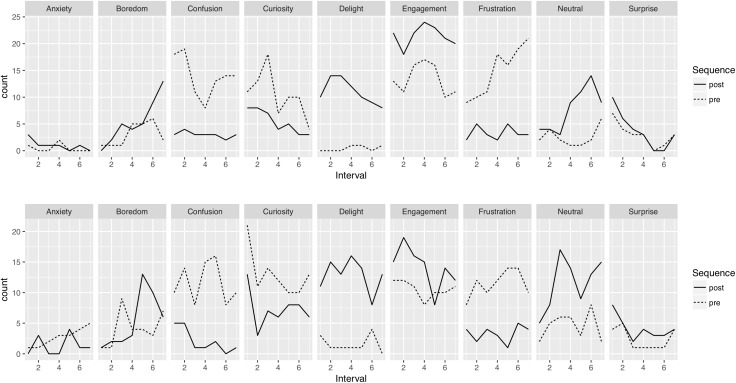
Counts of selection of the first emotion (upper row) and second emotion (lower row).

Because participants were asked to indicate every 1-min of the solving sequences the two most prevalent experienced emotions, the evolution of these data was expected to contribute to a better understanding of the dynamics of affective states that occurred during the solving sequences. Figure 6 shows the prevalence of the first emotions chosen along the seven intervals that were dividing the pre- and post-video sequences (upper row) and the prevalence of the second emotions (lower row).

A visual analysis of the graphics revealed a relative consistency between reports of the first emotion and the second one. Moreover, it seemed that the occurrence of some emotions evolved along the intervals. For example, Curiosity, Surprise tended to drop whereas some emotions such as Boredom, Frustration tended to increase during the sequences. However, watching the instruction video produced the most visible effect. When comparing the pre- and post-video sequences, clear patterns were visible, with for instance a diminution of confusion, frustration, and curiosity and an increase of delight, engagement, and neutral, in the post-video sequences. These observations were consistent the ones done with the network graph (see Figure 5) but showed the time progression of the selection of the emotions rather that the relationships between the first and the second emotions participants selected.

### Analysis of Solving Actions

Analyses were conducted in order to verify whether the solving behavior consisting of actions on the puzzles could reveal a relationship with the level of self-reported confusion. Because we assumed that solving activity was intrinsically different in the sequences preceding and following the watching of the instructional videos, we created a variable from of the average physical distance on the puzzles (expressed in pixels) between the coordinates of each click and the immediate preceding one. Based on the assumptions of the broader searching behavior of participants without a clear solving strategy, we expected the distance score to be larger in the pre-video than in the post-video sequences. However, the analyses did not reveal any significant difference on the variable distance between the pre- and the post-video sequences (*F* < 1). In other words, no evidence about any differentiated strategies according to the level of expertise of participants (manipulated by watching the videos) was found from the analysis of the average distance between clicks.

**FIGURE 7:**
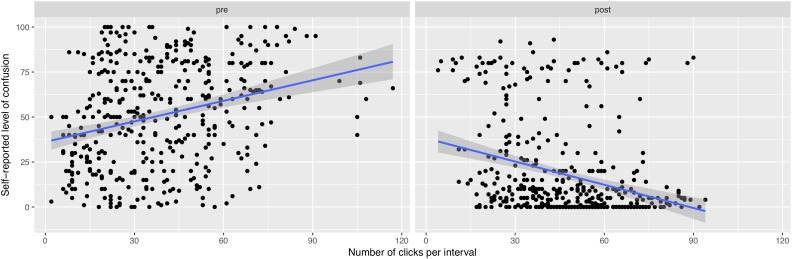
Relationship between the number of clicks per interval and the levels of self-reported confusion, before (left), and after (right) the instructional videos.

Another variable was considered with the total number of actions (clicks) participants produced on puzzles during each interval of the solving sequences. By matching these data with the levels of self-reported confusion for each interval, it was possible to observe differential trends in the relationship between these variables (see Figure 7). Because each participant generated seven points during each of the solving sequence, we calculated the correlation coefficients with a specific statistical technique allowing repeated observations (Bakdash and Marusich, 2017). During the pre-video sequences, a moderate positive correlation was observed between the level of confusion and the participants’ activity, *r_rm_*(402) = 0.446, *p* < 0.0001. However, in the solving sequences following the instructional videos, an inverse relationship was visible, with a moderate negative correlation between the variables, *r_rm_*(402) = -0.376, *p* < 0.0001.

Although the correlations were moderate, they showed the trend of a higher level of activity from participants who also reported higher levels of confusion before watching the video. Conversely, in the post-video sequences, the knowledge of a solving strategy produced the opposite trend: a diminution of confusion was here linked to a higher solving activity.

## Discussion

The primary aim of the study was to test whether the human-computer interaction could provide reliable indications on learners’ epistemic emotions, and notably their level of confusion, which can be particularly difficult to detect when occurring in digital environments (Arguel et al., 2017). To achieve this goal, a situation capable of generating participants’ confusion was required and we employed logic visual puzzles for creating a complex problem-solving task. The experimental material that we used was an adaptation of the electronic game Lights Out, which is based on logic puzzles, because it has been used in the past as an educational support to teach the mathematical technique of Gaussian elimination (Mulholland, 2011). Also, to ensure that a sufficient level of confusion was induced from the puzzles, it was required that participants were novice and lacked knowledge about effective solving strategies. We expected that using an electronic game dating from the 1990’s offered an unfamiliar experimental material to participants, which was verified since none of them knew the game prior to the study.

Involving novice participants was an important aspect of the study, supported by the assumption that a lack of prior knowledge should result in participants experiencing confusion when trying to solve puzzles. Because participants in our study had no experience with playing Lights Out games and had limited knowledge of the Gaussian elimination mathematical technique, they were all assumed novice. Hence, the difference of solving performance scores observed between the types of puzzle prior to watching the videos (M_Type-+_ = 0.74, M_Type-x_ = 0) was not considered as caused by a difference in prior knowledge. Some patterns of Type-+ puzzles were probably easier to solve intuitively compared with Type-x puzzles, which could explain the difference of performance. This assumption was consistent with the absence of significant difference of self-reported confusion between the types of puzzles. With both types of puzzle, the absence of usable knowledge about the strategy to employ in order to solve the puzzles was an obstacle to reaching the goal of the task. In this case, the source of confusion can be understood in terms of cognitive disequilibrium (Sullins and Graesser, 2014). Hence, we assumed that watching instructional videos depicting solving techniques should provide participants with usable how-to knowledge, which had as a consequence to improve their solving performance (i.e., the number of puzzles solved within the 7-min sequences) and also to decrease confusion. Our observations verified this assumption since the levels of confusion were significantly higher in the initial sequences than in the sequences following the instructional videos. In order to induce confusion at two separate occasions, two types of puzzles were used in the study (i.e., Type-x and Type-+). We specifically chose these types of puzzle because they required different solving strategies that could not be transferred between each other, contributing to prevent contamination between the conditions. When attempting to solve the second type of puzzle, it was hence anticipated that the inconsistency between the technique previously learnt and the results produced with the new type of puzzle should provoke confusion again. Consequently, we expected to observe in the pre-video sequence with second type of puzzle higher ratings of confusion associated with lower solving performance. Observations revealed that the level of self-reported confusion was indeed relatively high during all pre-video sequences, confirming that our experimental protocol was able to induce confusion twice, in a within-participants experimental design, with two types of puzzle. Moreover, to ensure that any potential training effect between the types of puzzle was neutralized for the analyses, their order of presentation was counterbalanced.

Despite being generally considered as a moderately negative emotion, confusion can sometimes be associated with benefits in terms of engagement of learners in the task (D’Mello et al., 2014). This quite counter-intuitive phenomenon was visible in our data when the participants were asked to choose, every 1-min interval, two emotions among a list of nine. In the pre-video sequences, the most frequently selected emotions, either as first or second emotion, were confusion, engagement, frustration, and curiosity. The juxtaposition of confusion with desirable emotions (i.e., engagement and curiosity) and an adverse emotion (i.e., frustration) was consistent with the model of affect dynamics that D’Mello and Graesser (2012) have described. The pivotal role of confusion occurring during cognitive activities has been represented with the image of a *zone of optimal confusion*, in which learners and problem-solvers navigate between positive and negative outcomes (Graesser, 2011; D’Mello et al., 2014). However, the interpretation of the causes of occurrence of emotions in our study needs to be taken cautiously. For instance, it is also possible that the positive emotions of engagement and delight were induced by satisfaction from an earlier episode of solving confusion, and not being actual independent co-existing emotions. Our argument is still speculative and it would be interesting to investigate, in future studies involving controlled experimental design, the impact of the exposition to transitory confusion – solved and unsolved – on subsequent emotional experiences.

While asking the participants to report at regular intervals the two most prevalent experienced emotions, the study generated a kind of snapshot of their emotional states every 1 min. In the post-video sequences, the level of self-rated confusion was lower than in the initial sequences but participants kept selecting this emotion, although to a lesser extent. Instead, the most cited emotions at post-video were engagement, delight, neutral, curiosity, and especially in the last intervals, boredom. The latter observation was not consistent with the *model of affect dynamics*, which gives, as explanation of the occurrence of boredom, an overwhelming confusion causing frustration, then boredom (D’Mello and Graesser, 2012). However, we assumed that the absence of confusion *per se* could also lead from engagement directly to boredom. Our data seemed to confirm this mechanism. Unlike in the D’Mello and Graesser’s study, confusion in our study was not induced at regular intervals from feedback provided by the environment, but by the task itself. When a new puzzle was presented to participants, they were prone to experience confusion until they could master an effective solving strategy, then, once able to apply this strategy, they tended to report positive emotions including engagement. However, from this stage, the lack of challenge and confusion to solve could potentially lead to experience a growing weariness, expressed by participants as boredom. This explanation is innovative because it suggests a new mechanism to explicate the benefits of being confused. Instead of considering submerging confusion as an indirect cause of boredom, it could actually be also the absence of confusion that led participants to progressively disengage from their activity while getting bored. Applied to education settings, this approach would suggest the benefits of a regular induction of confusion, even during the successful completion of tasks, in order to maintain learners’ engagement high and to prevent boredom.

Because the capability of detecting epistemic emotions, such as confusion, in real time is necessary to trigger adaptive educational interventions, our study also intended to explore the existence of potential links between participants’ behavior and their emotional responses during a problem-solving task. This objective was in line with the stream of research on the detection of epistemic emotions in digital learning environments (Calvo and D’Mello, 2010). A variety of methods have been tested in laboratory setting in order to provide reliable measurements of emotions, but one of the principal limitations of most of these techniques was the difficulty to implement them out of the lab, in real-world situations (Arguel et al., 2017). For this reason, priority was given to data that could be easily collected with existing computer terminals, without specific equipment. Indeed, even if eye-tracking equipment was used in our study, it was primarily to collect accurate data on time and locations of clicks, rather than to exploit the actual participants’ gaze trajectories. Indeed, collecting data from human-computer interaction would be relatively easy to achieve with the existing common hardware, thus we have considered this approach particularly promising for real-world applications.

Among epistemic emotions, we have brought a particular attention to confusion because of its potential impact on learning and problem-solving activities. In our study, we sought objective indicators of confusion from human-computer interaction. Based on previous research (Graesser et al., 2005), we hypothesized that, in the absence of substantial knowledge, solving strategies should have resulted in a broader exploration of the elements of the puzzles during the solving sequences and higher levels of self-reported confusion. To verify this hypothesis, we created a variable from the physical distance between successive clicks on puzzle during solving. Unfortunately, in our study this variable was not relevant for detecting confusion. In order to explain this absence of result, one can consider the differences existing between the original study and ours. For example, the material used (logical visual puzzles vs. technical drawing), the nature of the tasks (puzzle solving vs. breakdown scenario explanation), and the activity of participants (interaction with the puzzles vs. visual exploration). It is plausible that with a better-defined problem-solving task and a more controlled experimental environment, different solving strategies observable from the distance between clicks could have emerged from the noise of the data.

## Conclusion

Despite in our study considering the distance between successive clicks failed to produce conclusive results, we discovered that another indicator, namely the number of clicks on puzzle per interval, could actually be an indicator of confusion. Interestingly, the direction of the correlation between the number of clicks and confusion reversed with the effect of the instructional video. A positive correlation emerged prior to watching the videos, whereas the correlation became negative on sequences following the videos. This result was unexpected but might be interpreted in considering the difference of the task the participants had complete. Prior to watching the videos, the activity of participants while trying to solve the puzzles could be basically to explore the environment and to develop self-made solving strategies using a trial-and-error strategy. In the sequences following the videos, it is likely that the task rather consisted in retrieving and applying the strategy learnt previously from the video. Hence, the participants’ activity during the pre- and post-video sequences was probably different in nature and could have provoked confusion in two different ways. In the pre-videos sequences confusion would be linked to the failure of finding a satisfactory strategy, which was reflected by a larger number of actions. Inversely, in the post-videos sequences, confusion could be associated to a problem with applying a learnt strategy, hence producing a lower number of actions on the puzzles. Obviously, this assumption is hypothetical and would require replications with a finer analysis of the participants’ solving activities in order to confirm it. In future research, it will be relevant to control this variable, for example by collecting qualitative data about clicking behavior with the implementation of methodologies such as interviewing participants or using think-aloud protocol.

To conclude, in digital environments, the measurement of confusion can be difficult to achieve due to the variability of surrounding factors and the issue of adapting a measurement methodology with different tasks and environments. Moreover, the expression of epistemic emotions being highly variable among individuals, it can hinder the definition of generic indicators (Sullins and Graesser, 2014). Fortunately, the power of treatment of large datasets being nowadays immense, another approach could be involved. This approach would however require to code each participants’ interaction in order to treat all the available data as a whole, and then to train machine-learning algorithms to detect identified emotions during a cognitive task (Conati et al., 2013; DeFalco et al., 2018). Of course, the training of classifiers would probably require additional data collected from different sources, but the idea of compiling multimodal indicators of emotion could be a relevant way to achieve the development of reliable predictive models of confusion based on human-computer interaction (Wagner et al., 2011; Hussain et al., 2012). Further research is consequently needed with problem-solving tasks, but also with applications of any kind of cognitively demanding tasks, such as the learning of complex content.

## Ethics Statement

This study was carried out in accordance with the recommendations of the Australian Code for the Responsible Conduct of Research and the National Statement on Ethical Conduct in Human Research, with written informed consent from all subjects. All subjects gave written informed consent in accordance with the Declaration of Helsinki. Macquarie University Human Research Ethics and Integrity Committee formally approved the study protocol and the recruitment strategy (Approval No. 5201401132).

## Author Contributions

AA, LL, and MP contributed to conception and design of the study. KC programmed the experimental software. AA collected the data, performed the statistical analyses, and wrote the first draft of the manuscript. All authors contributed to manuscript revision, have read, and approved the submitted version.

## Conflict of Interest Statement

The authors declare that the research was conducted in the absence of any commercial or financial relationships that could be construed as a potential conflict of interest.
